# Medicare Payment Trends in Pulmonology and Critical Care (2003-2024)

**DOI:** 10.1016/j.chpulm.2026.100266

**Published:** 2026-05-23

**Authors:** David Brower, Arjun Bhatt, Nikhil Jaganathan, Shaheen Islam, William J. Healy

**Affiliations:** a Medical College of Georgia at Augusta University, Augusta, GA; b Department of Medicine, Medical College of Georgia at Augusta University, Augusta, GA

**Keywords:** critical care, health care economics, Medicare, pulmonology

## Abstract

**Background:**

Pulmonology and critical care physicians provide a diverse array of vital health services in a variety of settings. Reimbursement for these services is largely determined by the Centers for Medicare & Medicaid Services reimbursement schedule, which assigns relative value units (RVUs) to each service and adjusts it with a yearly conversion factor (CF) to determine reimbursement amounts.

**Research Question:**

How have changes in RVUs of specific services and the CF affected inflation-adjusted Medicare reimbursement change from 2003 to 2024 for pulmonology and critical care?

**Study Design and Methods:**

This study was a retrospective analysis using data from Medicare’s physician fee schedule. Inflation-adjusted facility and nonfacility Medicare rates were determined from each Current Procedural Terminology code using the Consumer Price Index. Total percent change was calculated for rate, CF, and component RVUs for each year from 2003 through 2024.

**Results:**

The fields of pulmonology and critical care faced substantial cuts in reimbursement from 2003 to 2024. Overall, nonfacility rates declined less than facility rates (−39.1 ± 22.8 vs −45.4 ± 16.4; *P* = .003) because of more favorable reimbursement changes for bronchoscopy in nonfacility settings (−31.3 ± 25.0 vs −45.8 ± 8.5; *P* = .009). There was no significant difference between declines in reimbursement for different practice locations, even though RVU components increased substantially more for bronchoscopy. The uniformity of the decline in reimbursement is mostly caused by cuts to the CF.

**Interpretation:**

Our results show that Medicare reimbursement has consistently declined relative to inflation because of sustained reductions in the CF across all practice locations. Nonfacility bronchoscopy reimbursement declined the least because of increased RVUs, but still declined 31.3% relative to inflation. Pulmonary and critical care medicine decline is in line with other specialties.

**Take-Home Points**

**Study Question:** How has the Centers for Medicare & Medicaid Services reimbursement changed on the Current Procedural Terminology code level for 4 pulmonary and critical care medicine (PCCM) practice locations from 2003 to 2024?

**Results:** Reimbursement for PCCM services was reduced considerably over the time period, falling 45% in the facility schedule and 39% in the nonfacility schedule.

**Interpretation:** This study shows that the decline in the conversion factor accounts for most of the reduction in reimbursement and explains why PCCM reimbursement trends are in line with other specialties.

Medicare cuts have concerned physicians in the US for decades.^1^ Because the Centers for Medicare & Medicaid Services (CMS) have long been a significant portion of the federal government’s budget, it has faced many reductions, and the American Medical Association has taken great effort to preserve reimbursement rates.^2^ Private insurance companies determine their reimbursement according to CMS standards^3^; therefore, decreases in CMS rates lead to lower reimbursement through the entire health care payer ecosystem. Thus, CMS reimbursement rates affect the financial stability of the county’s entire health care system.

Considering the importance of CMS rates on health care’s financial viability, many specialties have researched how changes have affected them specifically, especially since 2000. Dorius et al^4^ found that CMS reimbursement in otolaryngology rates has been cut nearly 30% from 2013 to 2024. Ophthalmology saw declines of > 33% from 2003 to 2024.^5^ Another study looked at the change in reimbursement for the 10 most commonly performed procedures of the 11 largest surgical specialties from 2000 to 2022, and found average reimbursement was cut −24.98% relative to inflation.^6^ Vascular surgery faced the largest cut of 43.05%, and neurologic surgery was reduced the least at 21.06%.^6^ There is ample evidence of reimbursement rate decline in surgical fields, but less work has been done in more cognitive specialties. Emergency medicine underwent nearly 30% reduction from 2000 to 2020,^7^ and some key procedures in cardiology also had inflation-adjusted decline.^8^ Gastrointestinal procedures also fell 33% relative to inflation from 2007 to 2022.^9^

Despite likely facing the same pressures as these fields, pulmonary and critical care medicine (PCCM) has not yet had their reimbursement rates rigorously examined. Comprehensive evaluation of longitudinal Medicare reimbursement trajectories, adjusted for inflation, for PCCM would provide a vital foundation for evidence-based changes in policy, health care accessibility, and sustainable medical care for a diverse and essential field of medicine.

The objective of this study is to examine Medicare reimbursement trends in PCCM across the following 4 practice locations: (1) bronchoscopy suite, (2) critical care units, (3) inpatient settings, and (4) outpatient settings. By analyzing historical trends in PCCM Medicare reimbursement and assessing the influence of conversion factors (CFs) and relative value units (RVUs), this study offers valuable insights into the future financial outlook of the field.

## Study Design and Methods

This retrospective study analyzed Medicare reimbursement trends in various practice settings for pulmonology and critical care physicians using publicly available data from 2003 to 2024. Because all data were publicly available and had no personal identifiable information, no institutional review board approval was required.

### Explanation of Medicare Reimbursement and Definitions of Key Terms

The CMS uses the Resource-Based Relative Value Scale, which assigns most Current Procedural Terminology (CPT) codes an RVU. RVUs are numerical values associated with a medical service, indicating the relative value of the service and resources required to provide such a service, allowing for quantitative comparisons of CPT codes. There are 3 components of the RVU: work RVU (wRVU), malpractice RVUs (mRVUs), and practice expense RVUs (peRVUs).^6^ There are 2 categories of peRVUs, including (1) facility rates, which include services by larger groups, typically associated with hospitals; and (2) nonfacility rates, which typically constitute private physician office services.^10^ It is important to note that facility rates are not synonymous with inpatient services; outpatient services provided by hospital operated groups (eg, pulmonology clinics owned by hospitals) are billed under the facility schedule under Point of Service 19 or 22. The RVU amount is then adjusted based on geography before being converted to a real dollar amount by the CF. The CF is a numerical dollar value ascribed by the CMS and is determined annually based on several factors, including the current economic state, quantity of Medicare beneficiaries, and specific directives of the CMS.^10^

### Delineation of Practice Settings

PCCM-trained physicians practice in a wide variety of settings. The 4 following practice settings were identified: outpatient clinics, which include sleep medicine; critical care units; inpatient services; and bronchoscopy, which also includes other pulmonology procedures (eg, thoracoscopy).

### Identifying CPT Codes

A total of 143 CPT codes relevant to PCCM were initially sourced from previous literature,^11^ resources from professional organizations,^12^^,^^13^ and individually by the practicing PCCM specialists and sorted into their appropriate practice location. From this initial list, only codes present throughout the entire 21-year study period were included for final analysis, reducing the total codes for final analysis to 69. In total, 25 codes were analyzed for bronchoscopy, 10 for critical care units, 8 for inpatient services, and 26 for outpatient clinics (e-Table 1).

### Data Collection and Key Calculations

For each selected CPT code, the following information was collected from the CMS Physician Fee Schedule from 2003 to 2024: wRVU, peRVU, mRVU, total facility and nonfacility RVU, procedure description, and CF. To adjust for inflation, the Consumer Price Index (CPI) data from January of each year between 2003 and 2024 were collected using the US Bureau of Labor Statistics.^14^ CMS reimbursement rates were calculated as the product of each year’s respective CF, and the total RVUs were calculated for the appropriate practice setting. No adjustment was made for Geographic Price Cost Index because this study’s focus is on national level trends, and the national average Geographic Price Cost Index is set to 1.0. Total percent change from the 2003 rate and inflation-adjusted rates were calculated as follows:

Unadjustedrate=totalRVUs×CF

Adjustedrate=unadjustedrate×CPI2003/CPIyear

Totalpercentchange=(CPTcodevariableyear/CPTcodevariable2003−1)×100%

For cumulative percent change comparisons between different practice locations, only the 2003 and 2024 rates were compared. In the longitudinal analysis of each location individually, each year from 2004 to 2024 was compared with the 2003 value to evaluate how reimbursement has changed over the 21 years of publicly available data. For all cases, the reported values represent the changes compared with the 2003 value, not annual growth rates.

### Statistical Analyses

The percent change of inflation-adjusted facility and nonfacility rates for each setting was determined from 2003 to 2004. Dunn’s test for multiple comparisons with the Bonferroni correction identified statistically significant changes between practice locations. Facility and nonfacility rates were compared using paired *t* tests. The longitudinal time analyses of inflation-adjusted facility rates, nonfacility rates, and RVU components reported running percent change from 2003. Facility vs nonfacility rates for each practice location were analyzed at the level of the specific CPT code. Statistical significance was set at α = 0.05 for individual comparisons and α < 0.017 for Bonferroni-corrected multiple comparisons. All statistical analysis was performed using Python (version 3.12).

## Results

**Figure 1:**
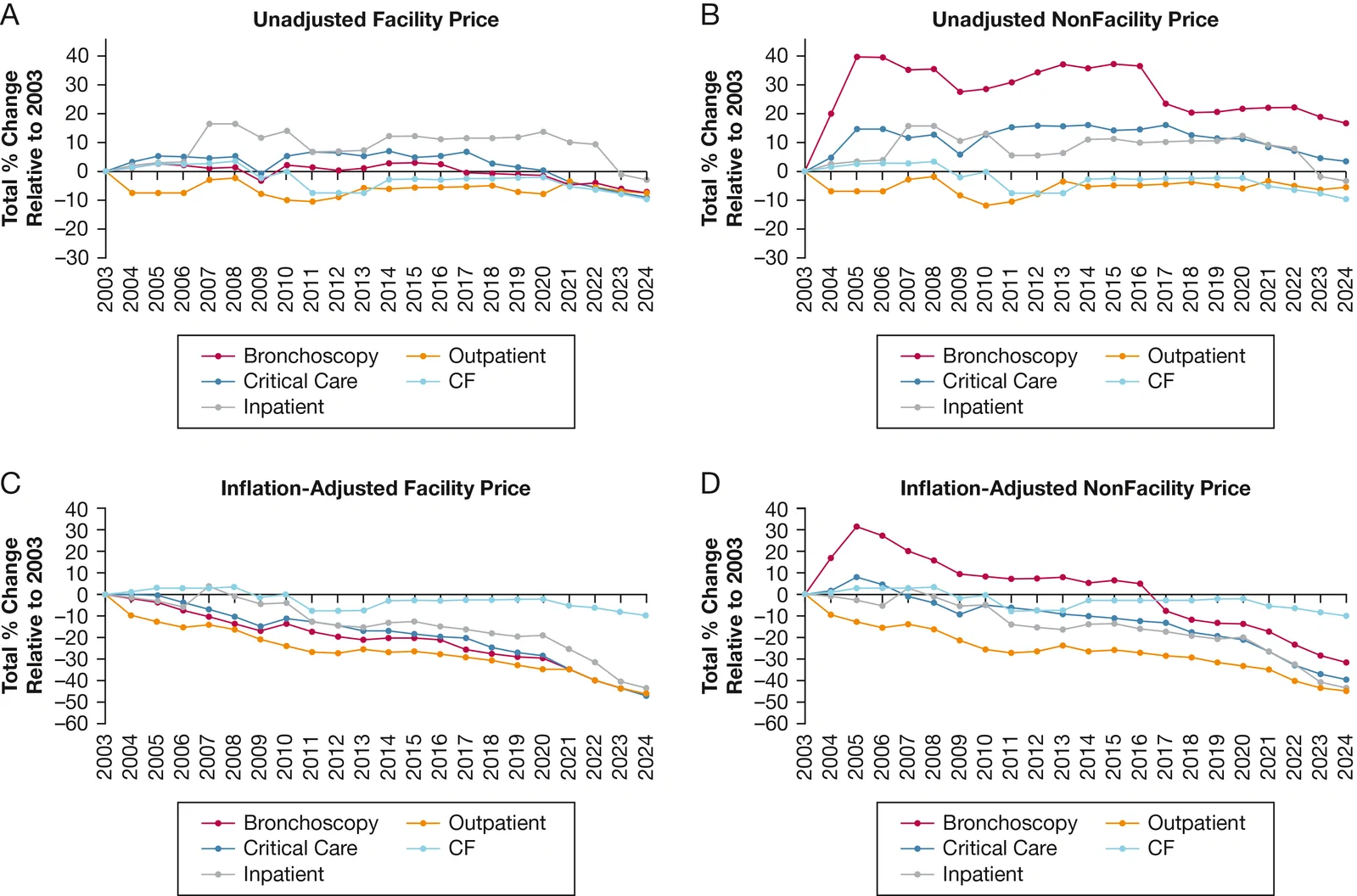
A-D, Changes in Medicare reimbursement rates in pulmonology, 2003 to 2024. Cumulative percent change in mean Medicare reimbursement for pulmonology practice locations from 2003 to 2024, stratified by setting (facility vs nonfacility) and inflation-adjusted rate. CF = conversion factor.

The unadjusted mean facility and nonfacility rate of each practice setting was plotted from 2003 to 2024 (Figs 1A, 1B). Unadjusted facility rates were largely stable over the study period, besides an increase in inpatient services rates in 2007 and a decrease in outpatient clinics in 2004 (Fig 1A). Likewise, unadjusted nonfacility rates were stable, excluding a 40% increase in bronchoscopy from 2003 to 2005 (Fig 1B). Relative to inflation, facility rates have steadily declined over the entire time frame, with no years of increase relative to inflation since 2015 for any location (Fig 1C). Nonfacility rates showed a similar consistent decline relative to inflation, apart from bronchoscopy increasing over 30% relative to inflation from 2003 to 2005. Facility reimbursement showed significantly greater decline than nonfacility (−45.4 ± 16.4 vs −39.1 ± 22.8; *P* = .003), largely driven by bronchoscopy’s lesser decline in nonfacility reimbursement rates (−45.8 ± 8.5 vs −31.3 ± 25.0; *P* = .009) (Table 1). No other practice setting had statistically significant differences in reimbursement rates between facility and nonfacility fee schedules. There was no significant difference in reimbursement reduction between all practice settings (Table 2).

**Table 1:** Inflation-Adjusted Mean Facility vs Nonfacility Rate Changes by Subspecialty, 2003 to 2024

Subspecialty	No.	Facility Rates	Nonfacility Rates	*P* Value
Bronchoscopy	23	−45.8 [8.5]	−31.3 [25.0]	.009
Critical care	9	−46.3 [9.3]	−39.3 [19.8]	.206
Inpatient	8	−43.1 [3.9]	−43.3 [3.1]	.711
Outpatient	26	−45.4 [24.4]	−44.6 [24.0]	.286
Total	66	−45.4 [16.4]	−39.1 [22.8]	.003

Data are presented as mean [SD] of cumulative percent change from 2003 to 2024 or as otherwise indicated. Statistical significance was determined using paired *t* tests comparing facility and nonfacility rate changes adjusted for inflation for the same Current Procedural Terminology codes within each practice location over the 21-y study period. Three codes (2 in bronchoscopy and 1 in critical care) are not included because they did not have a listed facility rate in either 2003 or 2004.

**Table 2:** Multiple Pairwise Comparisons by Subspecialty

Comparison	Facility Rates	Nonfacility Rates
	Mean % Δ	Mean % Δ
Bronchoscopy vs critical care	0.5	8
Bronchoscopy vs outpatient	−0.3	13.3
Bronchoscopy vs inpatient	−2.6	12
Critical care vs outpatient	−0.8	5.3
Critical care vs inpatient	−3.2	4
Outpatient vs inpatient	−2.3	−1.3

Pairwise comparison of mean percent change in rate adjusted for inflation from 2003 to 2004 across various practice settings is shown. *P* values were calculated using Dunn’s test with Bonferroni correction for multiple comparisons (n = 6). Statistical significance was defined as *P* < .00833. None of the comparisons were statistically significant (all *P* > .20).

**Figure 2:**
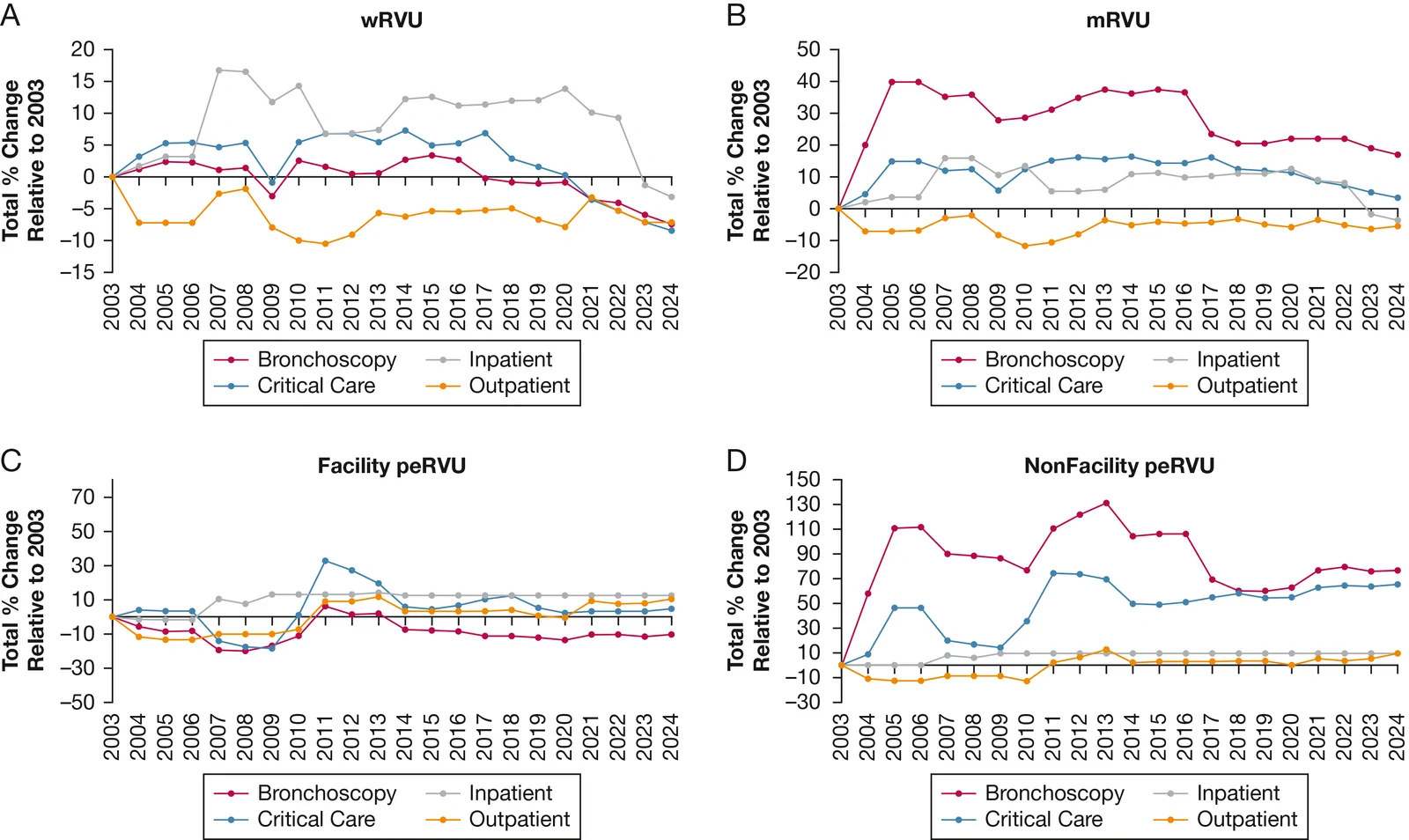
A-D, Changes in component relative value units in pulmonology, 2003 to 2024. Percent change in Medicare component relative value units for pulmonology practice locations from 2003 to 2024, relative to 2003 baseline, stratified by (A) wRVUs, (B) mRVUs, (C) facility peRVUs, and (D) nonfacility peRVUs. mRVU = malpractice relative value unit; peRVU = practice expense relative value unit; wRVU = work relative value unit.

Throughout the time period, RVU distribution remained stable. The wRVUs for inpatient services increased to 16.8% above 2003 levels but had fallen to –3% by 2024 (Fig 2A). The mRVUs for bronchoscopy increased by 40% over the initial 2 years, but fell considerably in 2017, eventually settling at a 17.1% increase over 2003 values at the end of the study period (Fig 2B). Facility peRVUs changed little, with the largest final change being a 12.9% increase in inpatient services. Nonfacility peRVUs saw the largest fluctuation, with bronchoscopy peaking at a 130.8% increase, before settling at a 76.8% increase in 2024 (Fig 2D).

Bronchoscopy reimbursement fared better in nonfacility settings. Diagnostic bronchoscopy with washes, brushes, and lavages (CPT codes 31622, 31623, and 31624) all fell by around 50% in facility settings, but their nonfacility rates fell considerably less, only being reduced to 37.6%, 29.7%, and 31.3%, respectively (e-Table 1). Bronchoscopy with biopsy and needle biopsy (CPT codes 31625 and 31629) increased 14.6% and 50.4%, respectively, relative to inflation in nonfacility settings but declined in facility billing. Critical care time (CPT codes 99291 and 99292) fell 38% relative to inflation in facility settings (e-Table 1). New office visits fell relative to inflation by at least 20% in both facility and nonfacility settings (CPT codes 99202-99205). Likewise, all established patient visits except for level 3 (CPT code 99213) decreased over the period.

## Discussion

Our study found a significant decline in Medicare reimbursement at all pulmonology practice settings over the 21-year period. Much of the decline was caused by the nearly 50% reduction in Medicare CF relative to inflation. The impact of these cuts was evenly distributed across all 4 of our practice settings. The facility schedule saw greater cuts than the nonfacility schedule, particularly in the bronchoscopy setting. Some bronchoscopy codes, such as CPT codes 31625 (bronchoscopy w/biopsy[s]) and 31629 (bronchoscopy/needle biopsy), each saw an increase in peRVUs in nonfacility settings, likely because of an explicit strategy from the RVS Update Committee to use increased practice expense to buoy reimbursement in the face of CF cuts outlined in their April 2002 minutes.^15^ Additionally, other codes, such as CPT 31623 (Diagnostic bronchoscope/brush), CPT 31628 (bronchoscopy/lung biopsy each), and CPT 31635 (bronchoscopy with foreign body removal), saw a much lower reduction than their facility counterparts. Despite this, nonfacility bronchoscopy still fell > 30% relative to inflation, highlighting how impactful the CF cuts have been.

Other work has previously illustrated trends in reimbursement in parts of PCCM (eg, critical care,^16^ sleep medicine^17^^,^^18^), but to date there has been no study that offers a comprehensive analysis of Medicare reimbursement trends across the entirety of PCCM at the CPT level. This study allows for the comparison of PCCM reimbursement decline with other specialties (e-Table 2). Although there is a difference in the years studied and methodology of selecting CPT codes, PCCM and gastroenterology seem to have declined roughly the same amount, whereas emergency medicine fared better than both (e-Table 2). Even when compared with the procedure-focused bronchoscopy subgroup, most surgical fields faced less severe declines than PCCM in general (e-Table 2, Table 1). Some of that difference is because of our selection of only codes that span the duration of our study period. Several new procedures in bronchoscopy (eg, endobronchial ultrasound) are compensated very highly by CMS but are not a part of our analysis, overestimating the decline in that practice location. There were no methodologically similar studies about adult primary care reimbursement, but declines should have been like the outpatient clinic group, considering office visit and other evaluation/management (E/M) codes are shared by all specialties. Our study shows that the PCCM CPT code level CMS reimbursement rate decline is roughly in line with other specialties; however, it may be more severe than most surgical fields. This alignment between different specialties is evidence that much of this erosion is caused by the decline of the CF.

Because the CF is uniformly applied to all Medicare spending, it serves to control the rate of growth of expenditure for the whole program. As Medicare faces a worsening budget crisis,^19^ lowering the CF remains a powerful yet blunt instrument for ensuring financial solvency for the program. CF reductions account for roughly 50% of the reduction in per patient spending^19^; however, as the Medicare population increases and ages, Medicare funding will be further stressed.^20^ Broad CF cuts will not be an effective strategy for maintaining the solvency of Medicare and supporting the country’s health care system indefinitely.

More deliberate spending adjustments have been attempted in recent years. Starting in 2021, the E/M code reimbursement was increased,^21^ and the CF was decreased to redistribute money to cognitive specialties.^22^ This change has had mixed effects on other specialties,^23^ but PCCM has not been specifically studied. All but 1 E/M code in our analysis (99213) saw decreased reimbursement over a 21-year period. Thus, despite an effort to prioritize cognitive reimbursement, cognitive codes are still compensated less in 2024 than in 2003.

This study has a few limitations. It only examined Medicare Part B fee-for-service claims and did not include Medicare Advantage or commercial insurance plans, which accounted for slightly over one-half of the 2024 Medicare market.^24^ Changes in coding practices and clinical guidance could have influenced our results, but the stability of RVU components suggests these effects are minimal. Additionally, we did not capture some new, highly reimbursed CPT codes in bronchoscopy, thus overestimating that practice location’s decline. Furthermore, because our study did not consider the volume of procedures performed, we could not look at changes in total CMS payments for each code. This may have obscured some technologic advances that increased the efficiency of a procedure, leading to it being done more quickly and often. Finally, our study cannot comment on changes in physician compensation because salary depends on a wide variety of factors beyond simple CMS reimbursement.

This study highlights the decline in Medicare reimbursement in PCCM. Future research should explore reforms that support the field’s financial stability to ensure adequate access to pulmonology and critical care services. We encourage PCCM physicians to participate in local and national groups to advocate for reimbursement protection. Physicians should also try to educate lawmakers and other elected officials on the deleterious effects of continuous CF cuts.

## Interpretation

Reimbursement for PCCM services has declined considerably across all practice settings in both facility and nonfacility schedules. RVU components have been largely stable across all settings. In contrast, the CF has decreased dramatically relative to inflation. These findings illustrate that the reduction in PCCM reimbursement is caused by the decline of the CF.

## Funding/Support

The authors have reported to *CHEST Pulmonary* that no funding was received for this study.

## Financial/Nonfinancial Disclosures

None declared.

## Supplementary Data

e-Online Data

e-Online Data
