# Supplementary material for: Medicare Payment Trends in Pulmonology and Critical Care (2003-2024)
Source: CHEST Pulm. 2026 May 23;4(3):100266. doi: 10.1016/j.chpulm.2026.100266 (PMC13562366; doi:10.1016/j.chpulm.2026.100266)
Supplement: Supplementary file 1 [file mmc1.docx]

**Supplementary Table 1:** Percent Change in Inflation-Adjusted Reimbursement by CPT Code, 2003-2024

| Subspecialty | CPT Code | CMS CPT Code Description | % ∆ in Facility Price | % ∆ in Non-Facility Price |
| --- | --- | --- | --- | --- |
| BS/OR | 31600 | Incision of windpipe | -54.5 | -54.5 |
| BS/OR | 31622 | Dx bronchoscope/wash | -49.1 | -37.6 |
| BS/OR | 31623 | Dx bronchoscope/brush | -50.6 | -29.7 |
| BS/OR | 31624 | Dx bronchoscope/lavage | -49.9 | -31.3 |
| BS/OR | 31625 | Bronchoscopy w/biopsy(s) | -49.7 | 14.6 |
| BS/OR | 31628 | Bronchoscopy/lung bx each | -48.9 | -19.3 |
| BS/OR | 31629 | Bronchoscopy/needle bx each | -39.1 | 50.4 |
| BS/OR | 31630 | Bronchoscopy dilate/fx repr | -49.3 | -49.3 |
| BS/OR | 31631 | Bronchoscopy dilate w/stent | -47.4 | -47.4 |
| BS/OR | 31635 | Bronchoscopy w/fb removal | -50.8 | -16.3 |
| BS/OR | 31640 | Bronchoscopy w/tumor excise | -49.8 | -49.8 |
| BS/OR | 31641 | Bronchoscopy treat blockage | -47.1 | -47.1 |
| BS/OR | 31645 | Brnchsc w/ther aspir 1st | -49.2 | -3.4 |
| BS/OR | 31646 | Brnchsc w/ther aspir sbsq | -43.7 | -43.7 |
| BS/OR | 31899 | Airways surgical procedure | 0 | 0 |
| BS/OR | 32400 | Needle biopsy chest lining | -45.6 | -27.5 |
| BS/OR | 32601 | Thoracoscopy diagnostic | -50.3 | -50.3 |
| BS/OR | 32604 | Thoracoscopy wbx sac | -48.3 | -48.3 |
| BS/OR | 32606 | Thoracoscopy w/bx med space | -48.3 | -48.3 |
| BS/OR | 32650 | Thoracoscopy w/pleurodesis | -42.9 | -42.9 |
| BS/OR | 32651 | Thoracoscopy remove cortex | -19.8 | -19.8 |
| BS/OR | 32653 | Thoracoscopy remov fb/fibrin | -21.6 | -21.6 |
| BS/OR | 32999 | Chest surgery procedure | 0 | 0 |
| BS/OR | 96570 | Photodynmc tx 30 min add-on | -47.6 | -48 |
| BS/OR | 96571 | Photodynamic tx addl 15 min | -49 | -49.7 |
| Critical Care | 36620 | Insertion catheter artery | -52.4 | -52.4 |
| Critical Care | 36625 | Insertion catheter artery | -41.2 | -41.2 |
| Critical Care | 36640 | Insertion catheter artery | -38.1 | -38.1 |
| Critical Care | 36660 | Insertion catheter artery | -44.7 | -44.7 |
| Critical Care | 76604 | US exam chest | -57.2 | -57.2 |
| Critical Care | 82803 | Blood gases any combination | 0 | 0 |
| Critical Care | 92950 | Heart/lung resuscitation cpr | -42.8 | 2.6 |
| Critical Care | 93503 | Insert/place heart catheter | -63.7 | -63.7 |
| Critical Care | 99291 | Critical care first hour | -38.4 | -24 |
| Critical Care | 99292 | Critical care addl 30 min | -38 | -35.2 |
| Inpatient | 99242 | Office consultation | -52.4 | -50.2 |
| Inpatient | 99243 | Office consultation | -43.5 | -43.6 |
| Inpatient | 99244 | Office consultation | -42 | -43.4 |
| Inpatient | 99245 | Office consultation | -41.1 | -43 |
| Inpatient | 99252 | Inpatient consultation | -41.4 | -41.4 |
| Inpatient | 99253 | Inpatient consultation | -39.8 | -39.8 |
| Inpatient | 99254 | Inpatient consultation | -41.8 | -41.8 |
| Inpatient | 99255 | Inpatient consultation | -43.1 | -43.1 |
| Outpatient | 93015 | Cardiovascular stress test | -59.5 | -59.5 |
| Outpatient | 94010 | Breathing capacity test | -59.7 | -59.7 |
| Outpatient | 94060 | Evaluation of wheezing | -67.1 | -67.1 |
| Outpatient | 94070 | Evaluation of wheezing | -80.1 | -80.1 |
| Outpatient | 94200 | Lung function test (mbc/mvv) | -69 | -69 |
| Outpatient | 94375 | Respiratory flow volume loop | -38.5 | -38.5 |
| Outpatient | 94621 | Cardiopulm exercise testing | -32.9 | -32.9 |
| Outpatient | 94660 | Pos airway pressure cpap | -43.3 | -30 |
| Outpatient | 94760 | Measure blood oxygen level | -61.4 | -61.4 |
| Outpatient | 94761 | Measure blood oxygen level | -71.1 | -71.1 |
| Outpatient | 94762 | Measure blood oxygen level | -50.8 | -50.8 |
| Outpatient | 95070 | Bronchial allergy tests | -75.7 | -75.7 |
| Outpatient | 95805 | Multiple sleep latency test | -64.4 | -64.4 |
| Outpatient | 95806 | Sleep study unatt&resp efft | -75.3 | -75.3 |
| Outpatient | 95807 | Sleep study attended | -53.6 | -53.6 |
| Outpatient | 95810 | Polysom 6/> yrs 4/> param | -53.3 | -53.3 |
| Outpatient | 95811 | Polysom 6/>yrs cpap 4/> parm | -52.7 | -52.7 |
| Outpatient | 99202 | Office o/p new sf 15-29 min | -40.1 | -32.3 |
| Outpatient | 99203 | Office o/p new low 30-44 min | -32.2 | -29.5 |
| Outpatient | 99204 | Office o/p new mod 45-59 min | -25.3 | -25.8 |
| Outpatient | 99205 | Office o/p new hi 60-74 min | -23.4 | -23.3 |
| Outpatient | 99211 | Off/op est may x req phy/qhp | -42.5 | -33.7 |
| Outpatient | 99212 | Office o/p est sf 10-19 min | -11.6 | -8.9 |
| Outpatient | 99213 | Office o/p est low 20-29 min | 10.6 | 4.2 |
| Outpatient | 99214 | Office o/p est mod 30-39 min | -0.4 | -5.8 |
| Outpatient | 99215 | Office o/p est hi 40-54 min | -8.2 | -9.6 |

**Supplementary Table 1 Legend:** Table reporting the percentage change in inflation-adjusted

Medicare facility and non-facility reimbursement for all 69 CPT codes using 2003 and 2024 dollar values. CPT codes showing a value of 0% (n=3) reflect procedures with no available facility price listed in either 2003 or 2024.
