# Supplementary material for: Medicare Payment Trends in Pulmonology and Critical Care (2003-2024)
Source: CHEST Pulm. 2026 May 23;4(3):100266. doi: 10.1016/j.chpulm.2026.100266 (PMC13562366; doi:10.1016/j.chpulm.2026.100266)
Supplement: Supplementary file 2 [file mmc2.docx]

| Field | Author | Date Range | Statistics | Result |
| --- | --- | --- | --- | --- |
| Pulmonology and Critical Care | Brower et al. (This study) | 2003-2024 | Mean Total % Change | -45.4 ± 16.4% Facility  -39.1 ± 22.8% Non-facility |
| Gastroenterology | Khunte et al. | 2007-2022 | Mean Total % Change | 33.0% (95% CI, −35.1% to −30.9%) |
| Ophthalmology | Bhatt et al. | 2003-2024 | Mean Total % Change | −33.5 ± 14.1 Facility  −38.9 ± 20.5 Non facility |
| Otolaryngology | Dorius et al. | 2013-2024 | Mean Total % Change | −29.9% (Standard Deviation 8.8) |
| Emergency Medicine | Pollock et al. | 2000-2020 | Mean Total % Change | -29% |
| Orthopedic Surgery | Singh et al. | 2000-2022 | Mean Total % Change | -25.1% |
| Neurological Surgery | Singh et al. | 2000-2022 | Mean Total % Change | -21.06% |
| Colon and Rectal Surgery | Singh et al. | 2000-2022 | Mean Total % Change | -23.68% |
| General Surgery | Singh et al. | 2000-2022 | Mean Total % Change | -24.95% |
| Obstetrics and Gynecology | Singh et al. | 2000-2022 | Mean Total % Change | -33.59% |
| Plastic Surgery | Singh et al. | 2000-2022 | Mean Total % Change | -24.54% |
| Thoracic Surgery | Singh et al. | 2000-2022 | Mean Total % Change | -37.92% |
| Urology | Singh et al. | 2000-2022 | Mean Total % Change | -33.29% |
| Vascular Surgery | Singh et al. | 2000-2022 | Mean Total % Change | -43.05% |
| Cardiac surgery | Singh et al. | 2000-2022 | Mean Total % Change | -28.01% |

Supplementary Table 2: Other similar studies studying change in CPT code level reimbursement in specific specialties.
